# Highly flexible and transparent colorless polyimide substrate sandwiched between plasma polymerized fluorocarbon and InGaTiO for high performance flexible perovskite solar cells

**DOI:** 10.1080/14686996.2024.2373041

**Published:** 2024-08-06

**Authors:** Su-Kyung Kim, Eun-Mi Cho, Hae-Jun Seok, Young-Yun Kim, Dong-Hyeok Choi, Sang-Jin Lee, Nam Joong Jeon, Han-Ki Kim

**Affiliations:** a School of Advanced Materials Science and Engineering, Sungkyunkwan University, Suwon-si, Gyeonggi-do, Republic of Korea; b Chemical Materials Solution Center, Korea Research Institute of Chemical Technology, Daejeon, Republic of Korea

**Keywords:** Colorless polyimide, plasma polymerized fluorocarbon, antireflective coating, InGaTiO, substrate, flexible perovskite solar cell

## Abstract

We integrated transparent antireflective coatings and transparent electrodes onto flexible colorless polyimide (CPI) substrates to fabricate high-performance flexible perovskite solar cells. Multifunctional PPFC/CPI/IGTO substrates were fabricated by sputtering the optimal plasma-polymerized fluorocarbon (PPFC) antireflective coating and InGaTiO (IGTO) electrode films on both sides of the CPI substrate. By applying PPFC with a low refractive index (1.38) as an antireflective coating, the transparency of the PPFC/CPI/IGTO substrate increased by an additional 1.2%. In addition, owing to the amorphous characteristics of the PPFC and IGTO layers, the PPFC/CPI/IGTO substrate showed constant sheet resistance and transmittance change even after 10,000 cycles during the bending tests. The flexible perovskite solar cells, fabricated on the PPFC/CPI/IGTO substrate, exhibited an increase in current density of 1.48 mA/cm^2^ after the deposition of the PPFC antireflective coating. These results confirmed that the PPFC/CPI/IGTO substrate was durable against high-temperature treatment, flexible, and exhibited excellent electrical characteristics. This enhanced the efficiency and durability of the flexible perovskite solar cells. Moreover, the hydrophobic PPFC layer allowed the self-cleaning of inflexible perovskite solar cells. Given these attributes, the PPFC/CPI/IGTO structure has been recognized as a good choice for multifunctional substrates of flexible perovskite solar cells, presenting the potential for enhancing performance.

## IMPACT STATEMENT

We have confirmed the durability of PPFC/CPI/IGTO substrates against high-temperature treatment, their flexibility, transparency, and their exceptional electrical properties, suggesting them as a prime selection for FPSCs.

## GRAPHICAL ABSTRACT

**Figure:**
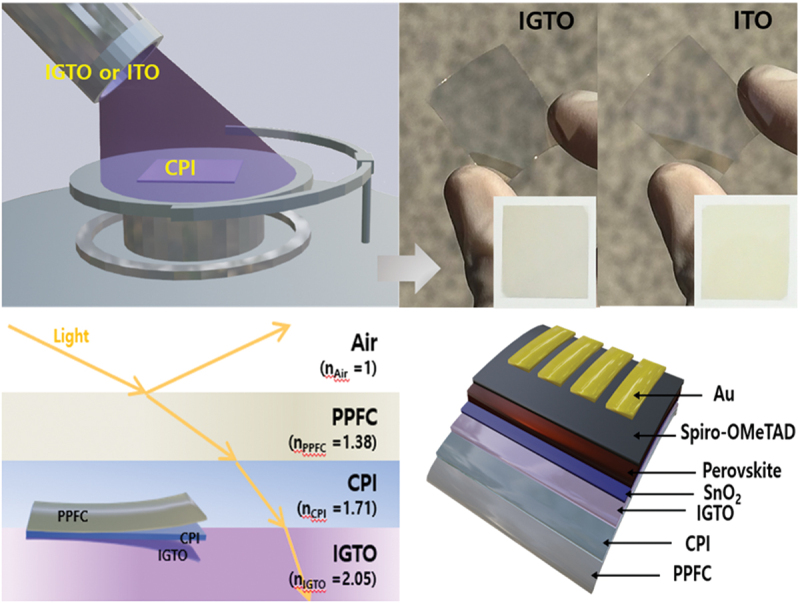

## 1. Introduction

Global warming is a pressing environmental concern, and next-generation photovoltaics offer clean and sustainable alternatives to traditional fossil fuel-based power generation. Among the emerging technologies in photovoltaics, perovskite solar cells have garnered significant interest, primarily owing to their remarkable power conversion efficiencies (PCE). Notably, the PCE of perovskite solar cells (PSCs) has experienced a substantial surge, reaching 25.7% in recent developments compared to the initial recorded value of 3.8% in 2009 [1–4]. In addition, perovskite solar cells have advantages as a flexible solar cell, such as low cost, simple device structure, bending durability, light weight, and a low temperature process [5–7]. Flexible perovskite solar cells (FPSCs) have been extensively investigated owing to their potential applications in portable electronics, bendable display devices, and wearable electronic textiles [6–12]. In general, FPSCs are fabricated by depositing electrodes, charge-transport layers, and perovskite active layers on flexible, transparent substrates. A flexible substrate mechanically supports a solar cell and protects it from the environment, such as the atmosphere. Flexible substrates include metal-, ceramic-, and polymer-type substrates. Polymer substrates have received attention as FPSC substrates because they are lightweight, inexpensive, and highly flexible [13,14]. Polyethylene terephthalate (PET) and polyethylene naphthalate (PEN) are the most commonly used transparent polymer substrates for FPSCs. Although PET and PEN exhibit outstanding optical transparency, their susceptibility to low glass transition temperatures (PET T_g_: ~78°C, PEN T_g_: ~123°C) poses a challenge in the fabrication process. Consequently, both the optical and mechanical properties of these polymers deteriorate when they are exposed to elevated temperatures during the fabrication process [14–16]. In comparison, colorless polyimide (CPI) has high temperature stability with a T_g_ of about 300°C, thermal decomposition temperature (T_d_) of approximately in the range of 350 to 570°C and excellent dimensional stability [14–16]. While the fabrication temperature of PSCs has reduced, materials such as TiO_2_, commonly employed as the electron transport layer (ETL) for PSCs, are deposited at temperatures surpassing 450°C [17,18]. In addition, materials used for hole transport layer (HTL), such as NiO_x_ thin film [19–21] and PEDOT:PSS (poly(3,4-ethylenedioxythiophene):poly (styrenesulfonic acid)) [22,23], require high-temperature annealing processes. The thermal stability of a flexible substrate is crucial for its suitability as a substrate for PSCs. As CPI has excellent transparency and dielectric properties, it is a suitable flexible substrate for PSCs. Flexible electrodes deposited on flexible substrates also significantly affect the PCE and mechanical properties of flexible solar cells [24]. Indium tin oxide (ITO) is the most commonly used electrode for transparent conductive oxide (TCO) in flexible solar cells. Although ITO has a high conductivity and optical transparency, its crystalline properties can result in severe crack formation and propagation. To overcome this limitation, amorphous TCOs have been extensively studied because of their outstanding mechanical flexibility. Among the many amorphous TCOs, amorphous indium-gallium-titanium oxide (IGTO) has been employed in flexible solar cells owing to its high flexibility, conductivity, and transparency, making it a promising alternative to typical ITO electrodes [25–27]. In addition, to increase the efficiency of FPSCs, the application of an antireflective coating (ARC) under the substrate to help absorb photons has been considered. To mitigate the Fresnel reflection resulting from the disparity in refractive indices between air and the substrate, materials with refractive indices intermediate between those of air and the substrate can be employed to effectively diminish reflection [28]. ARCs are of many types, including single-layer, multilayer, and patterned films [29]. Among them, single-layer ARCs have the advantages of a simple production process, low cost, and relatively high durability compared with multi-layer ARCs. Plasma polymer fluorocarbon (PPFC) thin films, which are used as single-layer ARCs, exhibit a low refractive index, high hydrophobicity, transparency, electrical stability, flexibility, thermal stability, and high surface hardness [30–34]. Cho *et al*. reported a 1.4% enhancement in the average transmittance in the visible-light region by applying PPFC as an ARC film in perovskite solar cells with a PET substrate, leading to a 1.8% increase in the PCE [32]. Although the individual characteristics of the IGTO electrode and PPFC ARC films have been extensively documented, there is currently a gap in the research concerning integrated CPI substrates with both IGTO and PPFC (PPFC/CPI/IGTO). Considering the characteristics of CPI, IGTO, and PPFC, it is inferred that PPFC/CPI/IGTO substrates will exhibit high flexibility, conductivity, and transparency, and they also have the capability for anti-reflection. The PPFC/CPI/IGTO substrates with these characteristics can be utilized not only in solar cells but also in various electronic components such as OLEDs (Organic Light Emitting Diodes), LCDs (Liquid Crystal Displays), electrochromic devices, and smart windows [35–37].

In this study, we integrated an amorphous IGTO electrode and a PPFC ARC layer on top of and under a flexible CPI substrate to obtain high-performance FPSCs. Using the optimized IGTO and PPFC ARC layers, we fabricated a multifunctional flexible PPFC/CPI/IGTO substrate at room temperature. The mechanical and optical properties of CPI as a flexible substrate were assessed, and the electrical, optical, and flexibility characteristics of IGTO as a flexible electrode were evaluated. Finally, the optical properties and device-protection capabilities of the PPFC as an ARC were investigated to confirm its potential applicability. To demonstrate the feasibility of the PPFC/CPI/IGTO substrate, we fabricated FPSCs on IGTO/CPI with or without the PPFC ARC layer and compared their performances.

## 2. Experimental section

### 2.1. Preparation and characterization of flexible electrodes

To deposit flexible IGTO electrodes, a 50-μm-thick, flexible CPI substrate was prepared. To deposit the IGTO electrode, we used a 4-inch IGTO target (Advanced Nano Products Co., Ltd.), which was 1 wt% Ga and 1 wt% Ti co-doped In_2_O_3_. The sputtering process was performed in an optimal environment with 3.3 × 10^−7^ m^3^/s of Ar, a working pressure of 3 mTorr, a DC power of 40 W and deposition rate 7.1 × 10^−11^ m/s. For the reference ITO electrode, we used an ITO target with 10 wt% Sn-doped In_2_O_3_. The reference ITO films were sputtered in an Ar environment at 3.3 × 10^−7^ m^3^/s, a working pressure of 3 mTorr, a DC power of 100 W and deposition rate 17.5 × 10^−11^ m/s. To determine the most suitable sputtering power for ITO and IGTO, electrodes were fabricated with power as the variable, and their characteristics were evaluated. It was found that for IGTO, the highest figure of merit (FOM = transmittance^10/sheet resistance) was achieved with 40 W power, whereas for ITO, the highest FOM was observed with 100 W power. Thus, deposition was conducted with each respective power. During the sputtering process, the substrate was rotated at 15 rpm to achieve electrode uniformity and all the electrodes were deposited with a thickness of 150 nm. Furthermore, to maintain the amorphous characteristics of the electrodes and increase flexibility, they were fabricated at room temperature. To confirm the electrical properties and determine the work functions of the electrodes, Hall effect measurements (HMS-4500AM, Ecopia, Republic of Korea) and ultraviolet photoelectron spectroscopy (UPS; Sigma Probe, Thermo VG Scientific, USA) were performed. For optical and surface examinations, UV-visible spectroscopy (V-670, Jasco, Japan) and field-emission scanning electron microscopy (FE-SEM, JSM-7600 F, JEOL, Japan) were employed.

### 2.2. Preparation and characterization of antireflective coating layer

The PPFC layer was deposited on the CPI substrate using a mid-range frequency (MF) sputtering process. The carbon nanotube (CNT) – perfluoroalkoxy (PFA) composite target used for this deposition was composed of 5 wt% CNT (K-nanos 200P, Kumho Petrochemical) and 95% PFA (6503PBZ, Dyneon). MF sputtering was performed at a working pressure of 8 mTorr and sputtering power of 300 W. PPFC was deposited on three samples with thicknesses of 70, 110, and 150 nm by adjusting the deposition time. X-ray photoelectron spectroscopy (XPS, K-alpha, Thermo Scientific, U.K.) was conducted to verify the chemical bonding of the PPFC layer. Additionally, spectroscopic ellipsometry (V-VASE, J. A. Woollam, USA) and UV-visible spectroscopy (V-670, Jasco, Japan) were conducted to investigate the optical properties. The surface morphology was analyzed using field-emission scanning electron microscopy (FE-SEM, JSM-7600 F, JEOL, Japan) and atomic force microscopy (AFM, NX-10, Park Systems, Republic of Korea). The water contact angle (WCA) was calculated using a contact angle analyzer (Phoenix-MT(A), SEO Co., Republic of Korea).

### 2.3. Fabrication and characterization of flexible perovskite solar cell

To enhance the wettability of the SnO_2_ ETL deposited through the solution process, the PPFC/CPI/IGTO samples were subjected to UV-ozone treatment for 60 min. A solution containing SnO_2_ nanoparticles (Alfa Aesar, 20 wt% in deionized water) was diluted to four times the volume of deionized water and deposited by spin-coating. During the spin-coating process, the substrate was rotated at 3,000 rpm for 30 s, and annealing was performed at 100°C for 30 min after spin-coating. A solution of 5 mM glycidyltrimethylammonium chloride (Aldrich) in 2-propanol was spin-coated at 5,000 rpm for 30 s on the SnO_2_ layer. Subsequently, annealing was performed at 100°C for 1 h. To deposit the perovskite layer, a mixed solvent of dimethylformamide and dimethyl sulfoxide in a volume ratio of 8:1 with a total volume of 0.9 mL was prepared. A solution was prepared by dissolving 1.4 M FAPbI_3_, 0.5 M methylamine hydrochloride, and 0.07 M MAPbBr_3_ (Aldrich) in 0.9 mL of the solvent. The perovskite active layer was spin-coated using this solution under the condition that it was processed at 1,000 rpm for 5 s and then at 5,000 rpm for 20 s. Before the rotation was completed, 1 mL of diethyl ether, as an antisolvent, was dropped over 5 s. After the spin-coating process was completed, annealing was carried out at 100°C for 1 h. Spiro-OMeTAD (LumTec) was used as the HTL and deposited using a spin-coating process. Spiro-OMeTAD (0.91 g) was dissolved in 1 mL of chlorobenzene. To this solution, 21 μL of bis(trifluoromethane)sulfonimide lithium (540 mg/mL in acetonitrile), 9 μL of FK209 (376 mg/mL in acetonitrile, tris(2-(1 H-pyrazol-1-yl)-4-tert-butylpyridine)cobalt(III) tri[bis(trifluoromethane)sulfonimide]), and 35 μL of tert-butyl pyridine were added to prepare the solution for spin-coating. Spin coating was performed at 2,000 rpm for 30 s. Finally, Au was used as the top electrode and deposited via thermal evaporation under a vacuum environment at 5 × 10^−5^ Torr. To evaluate the photoelectric conversion properties of the fabricated solar cells, J-V curves were obtained using a solar simulator (Oriel Class A, 91195A, Newport) and a voltage source meter (2420 Instruments, Keithley). In addition, external quantum efficiency (EQE) spectra were measured. A 450-W xenon lamp (Oriel) with an AM 1.5 G filter was used as the light source. Cross-sectional images of the device were obtained using high-resolution transmission electron microscopy (HR-TEM).

## 3. Results and discussion

**Figure 1.**
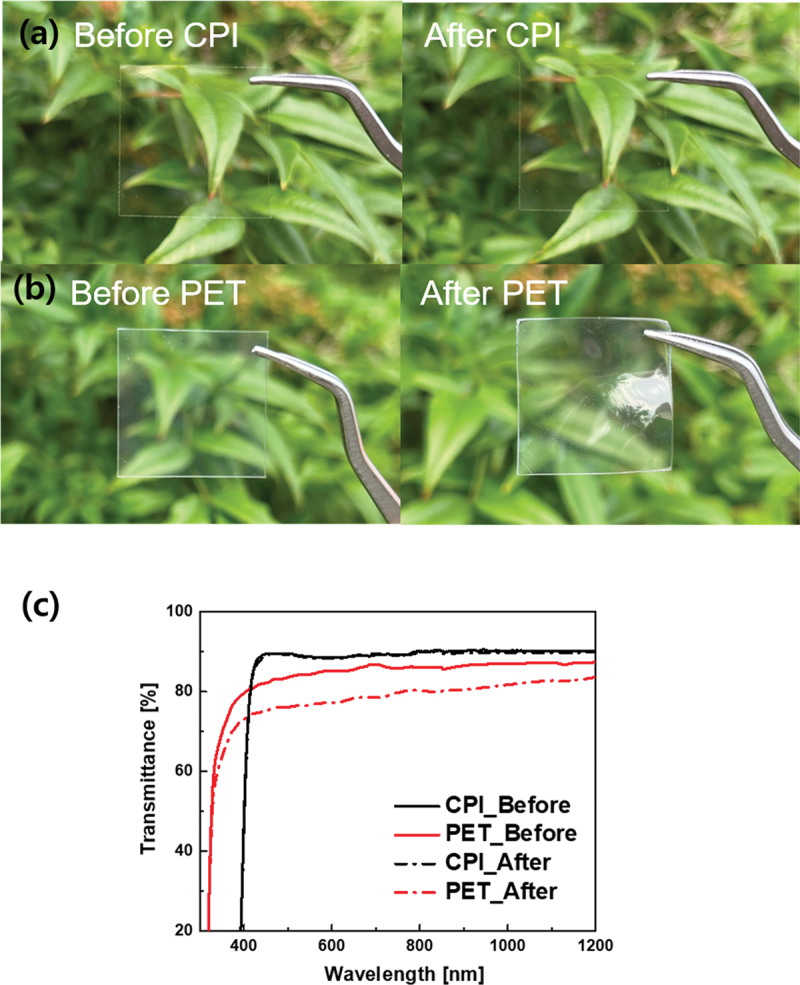
Photographs of (a) CPI and (b) PET substrates before and after heat treatment at 180°C for 10 min. (c) Optical transmittance change of the CPI and PET substrates before and after heat treatment.

Figures 1(a,b) show the photographs of the flexible CPI and PET substrate before and after thermal heating at 180°C for 10 min. In the case of the CPI substrate, the sample showed a similar transmittance without degradation owing to the high thermal stability of the CPI substrate, with T_g_ exceeding 300°C [16]. However, typical PET substrates undergo deformation and transmittance changes after heat treatment. In general, PET has a low T_g_ of approximately 78°C; therefore, high-temperature processes above 100°C can lead to changes in its optical and mechanical properties. PET is not a suitable flexible substrate for the fabrication of flexible PSCs. Figure 1(c) shows the optical transmittances of the CPI and PET substrates before and after the heat treatment. And the optical properties of PEN before and after heat treatment, commonly used for flexible substrates, are shown in Figure S1. The transmittance of the CPI substrate rapidly increased near 400 nm, whereas PET exhibited a higher transmittance than CPI at wavelengths below 400 nm. However, in the visible range of 400–800 nm, CPI before the heat treatment showed a higher average transmittance (88.8%) than PET (84.7%). Even at 550 nm, the transmittance of the CPI was 88.4%, which was higher than that of PET (84.3%). Because most commercialized CPI contains a trifluoromethyl (−CF3) structure, it exhibits high thermal stability and optical transmittance characteristics [14]. Even after heat treatment, the CPI maintained a high average transmittance of 88.3% in the wavelength range of 400–800 nm. However, for PET, the average transmittance significantly decreased to 77.0%, indicating damage to durability caused by heat. To produce perovskite solar cells, heat treatment at temperatures exceeding 100°C is required [17–23]. Therefore, as CPI films maintain high durability even after high-temperature processing, they are suitable substrates for perovskite solar cells. And the PPFC, considered as an anti-reflective coating, also shows no changes in chemical bonding state even after heat treatment at 300 degrees, and indicating high thermal stability as it maintains over 90% transmittance and a water contact angle value of over 100 degrees [33].

**Figure 2.**
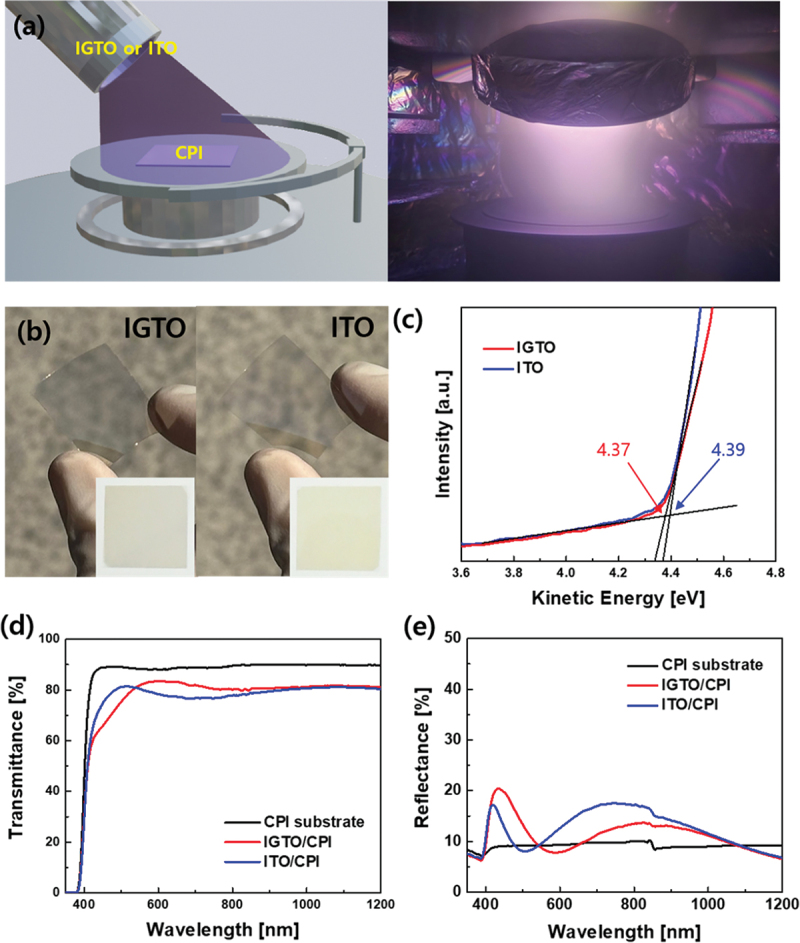
(a) Schematic and photograph of the sputtering process of the IGTO electrode deposition onto the CPI substrate. (b) Picture of the flexible IGTO and ITO electrodes on the CPI substrate. (c) UPS spectra showing the energy band levels of the IGTO and ITO electrodes on the CPI substrate. (d) Optical transmittance and (e) reflectance spectra of IGTO or ITO on the CPI substrate.

Figure 2(a) shows a schematic and a picture of the room-temperature sputtering process for depositing a flexible IGTO electrode on a CPI substrate. Using a tilted cathode gun, a high-quality IGTO electrode was deposited on a CPI substrate without heating the substrate. Figure 2(b) shows the flexible IGTO and reference ITO electrodes coated onto a CPI substrate. To compare the work functions of the flexible IGTO and ITO electrodes, UPS was performed, as shown in Figure 2(c), which showed that the work function (4.37 eV) of the IGTO film was similar to the work function (4.39) of typical ITO films. The similar work function of IGTO indicates that IGTO electrodes are promising flexible transparent electrode (FTE) substitutes for brittle ITO electrodes, as reported in our previous work [25–27]. Table 1 compares the electrical characteristics of IGTO and ITO electrodes on the CPI substrate.

**Table 1.** Electrical properties of IGTO and ITO flexible electrode on CPI.

Electrode	Sheet resistance [ohm/square]	Resistivity [×10^−3^ ohm-cm]	Mobility [cm^2^/V-s]	Carrier Con. [×10^19^/cm^3^]
IGTO	28.2	0.42	8.04	184
ITO	69.9	1.05	6.76	88

In general, a decrease in oxygen vacancies leads to a reduction in the carrier concentration of InO electrodes. However, this can be compensated by doping them with Ti. By substituting the In^3+^ ions with Ti^4+^ ions, additional charges are introduced into the In_2_O_3_ matrix. Moreover, the electrophilic properties of Ti reduce carrier scattering, preventing a decrease in mobility despite an increase in the carrier concentration [25,26]. ITO deposited at temperatures below 170°C, which is the crystallization temperature of ITO, results in amorphous characteristics, offering higher flexibility compared to crystalline ITO [38]. However, amorphous ITO has high resistance owing to its low mobility and carrier density [38]. In contrast, IGTO deposited at room temperature is amorphous, providing flexibility while also having a high carrier density and mobility, as explained earlier regarding the role of Ti^4+^ ions. Therefore, it can be inferred that the electrical properties of IGTO are superior to those of ITO.

Figures 2(d,e) show the optical characteristics of the flexible IGTO and ITO electrodes deposited on the CPI substrate. In the range of 400–540 nm, ITO exhibited a higher transmittance. However, in the range of 540–1200 nm, the transmittance of IGTO was higher. The overall average transmittances at 400–800 nm and 550 nm were 76.4% and 81.0% for IGTO, and 76.1% and 80.2% for ITO, respectively. In addition, IGTO exhibits better transmittance in the visible light spectrum. This is because of the influence of the doped Ga and Ti, which cause IGTO to possess amorphous characteristics and reduce oxygen vacancies, thereby reducing obstacles to light scattering [26,27,29,30]. Figure S2 shows the variation in the refractive indices of IGTO and ITO as a function of wavelength. The average refractive index of IGTO and ITO between 400 and 800 nm was 2.06, showing no difference; their refractive index at 550 nm was also similar at 2.08. However, the difference between the maximum and minimum values of the refractive index was smaller for IGTO, exhibiting only a small variation in its values in the 400–800 nm range. Generally, perovskite solar cells are known to absorb light in the wavelength range of 400–800 nm, exhibiting the photovoltaic conversion effect [39]. Therefore, considering the optical properties, IGTO is more suitable as an electrode than ITO, as it can absorb light within the absorption wavelength range of perovskite solar cells into the solar cell.

**Figure 3.**
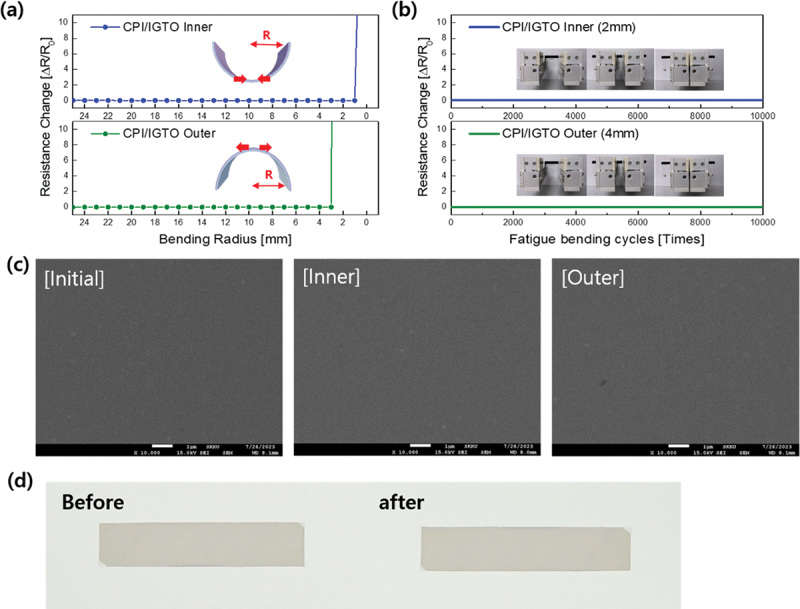
(a) Resistance changes of flexible IGTO on CPI in outer and inner bending tests. The inset indicates the bending radius. (b) Repeated inner and outer bending tests at fixed bending radii of 2 and 4 mm repeated 10,000 times. (c) Surface SEM images of initial IGTO and after inner and outer fatigue test performed 10,000 times. (d) Picture of IGTO/CPI samples before and after fatigue test.

A bending test was performed using lab-made inner and outer bending testers to confirm the flexibility of the IGTO electrode on the CPI substrate. Figure 3(a) shows the change in resistance of the CPI/IGTO flexible electrode as a function of the bending radius. When the IGTO/CPI sample underwent inner bending, a compressive stress was applied to the IGTO electrode. Conversely, tensile stress was exerted on the IGTO electrodes during the outer bending of the IGTO/CPI sample, as illustrated in the inset. With decreasing bending radius, the IGTO/CPI sample showed no change in the in situ measured resistance. However, at the inner and outer bending radii of 2 and 4 mm, respectively, the sample showed an abrupt increase in resistance. Therefore, the critical inner and outer bending radii of flexible IGTO were considered to be 2 and 4 mm, respectively. In addition, to verify the repeated durability of the bending characteristics, a fatigue bending test was conducted for 10,000 cycles based on the critical inner radius of 2 mm and outer radius of 4 mm. The resistance maintained its initial value for up to 10,000 cycles, indicating the outstanding flexibility of the IGTO electrodes. The changes in the surface morphology of IGTO were examined using SEM before and after the IGTO electrode was subjected to a repeated bending test of 10,000 cycles, as depicted in Figure 3(c). Remarkably, even after 10,000 cycles, no cracks or defects were observed, irrespective of the bending mode. Figure 3(d) shows the photographs of the IGTO/CPI samples before and after the fatigue test. Consistent with the SEM findings, the samples exhibit identical images without any signs of degradation.

This demonstrates the excellent characteristics of the IGTO/CPI electrode as a flexible electrode for solar cells.

**Figure 4.**
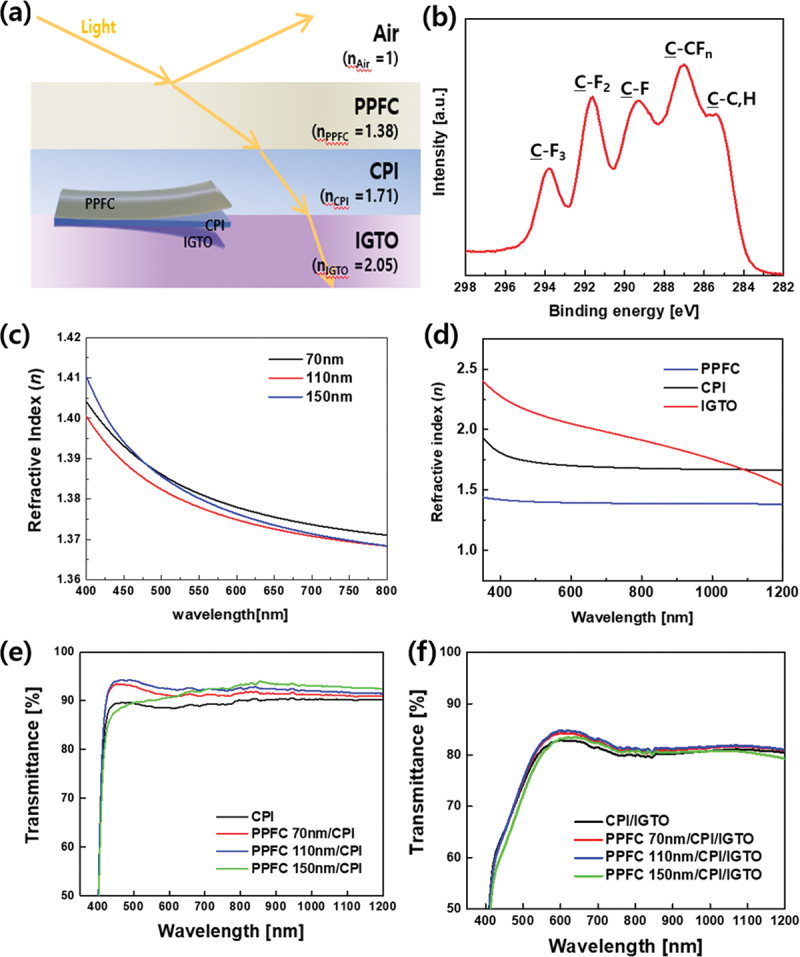
(a) Schematic of the antireflection effect in the PPFC/CPI/IGTO substructure of FPSCs. (b) XPS spectrum of PPFC. (c) Variation of refractive index of PPFC with film thickness. (d) Refractive index of each substructure. (e) Optical transmission spectra of PPFC and (f) PPFC on CPI/IGTO.

To enhance the efficiency of the FPSCs, PPFC was deposited as an antireflective coating layer on the opposite side of the CPI substrate where the electrodes would be deposited, as shown in Figure 4(a). Figure 4(b) shows the XPS data analyzed for the chemical bonding of the sputtered PPFC and the C 1s spectra. The spectra show peaks corresponding to C-F_3_ (293.8 eV), C-F_2_ (291.6 eV), C-F (289.3 eV), C-F_n_ (287.0 eV), and C-(C, H) (285.3 eV) caused by impurities from surface adsorption [30]. The C-F_x_ functional groups have a low surface energy, making them highly stable against reactions in external environments [14], and they exhibit a low refractive index and excellent electricity and heat insulation properties [30]. In particular, trifluoromethyl (-CF_3_) contributes to increased thermal stability and light transmittance [15,16]. As shown in Figure 4(a), light enters the solar-cell device from the air through PPFC, CPI, and IGTO. The reflection of light occurs according to the change in the refractive index, which is a characteristic of the material, when the medium through which light travels changes. When an ARC layer is applied, the reflectance R of light is defined by Equation (1) [29].

(1) $$R={\left[\frac{n_{\mathrm{air}}\;n_{\mathrm{sub}}-n_{ARC}^{2}}{n_{\mathrm{air}}\;n_{\mathrm{sub}}+n_{ARC}^{2}}\right]}^{2}$$

$\mathrm{where}\;n_{\mathrm{air}}$, $n_{\mathrm{sub}}$, and $n_{\mathrm{ARC}}$ represent the refractive indices of the air, substrate, and ARC, respectively. To minimize reflection, $n_{\mathrm{air}}\;n_{\mathrm{sub}}=n_{ARC}^{2}$ must be satisfied. When using a CPI substrate, as shown in Figure 4(a), with $n_{\mathrm{sub}}$ = 1.71, $n_{\mathrm{air}}=1$, the optimal refractive index value for the ARC layer is 1.31.

However, materials with low refractive indices do not exist. Therefore, research is being conducted to lower the refractive index of ARC layers. To investigate the variation in the refractive index according to the PPFC thickness, PPFC layers with thicknesses of 70 nm, 110 nm, and 150 nm were deposited, as shown in Figure 4(c), and their respective refractive indices were measured. While the refractive index values did not show significant differences based on thickness, the 110 nm thickness exhibited the lowest average refractive index of 1.377 within the 400–800 nm range, as shown in Figure 4(c). At a wavelength of 550 nm, the refractive indices for PPFC thicknesses of 70 nm, 110 nm, and 150 nm were 1.381, 1.377, and 1.380, respectively, indicating similar values. The thickness (d) of the ARC that minimizes reflection can be expressed as shown in Equation (2) [40,41].

(2) $$d=\frac{\mathrm{m\lambda}}{4n_{\mathrm{ARC}}}$$

where d is the thickness of the ARC, m is an odd number, λ is the wavelength of the incident light, and n_1_ is the refractive index of the antireflection film. By substituting the refractive index of the PPFC at a wavelength of 550 nm in Equation (2), an optimal thickness of approximately 100 nm is obtained. When PPFC with a thickness of 110 nm, which is relatively close to the optimal value, was deposited on the CPI, it exhibited the highest transmittance at 550 nm compared to PPFC at thicknesses of 70 nm and 150 nm, as shown in Figure 4(e). In the visible range of 400–800 nm, the average transmittance of the sample with a 110-nm-thick layer of PPFC deposited on the CPI increased to 91.7%, compared to the bare CPI transmittance of 88.0%. Samples with PPFC films with thicknesses of 70 nm and 150 nm also exhibited transmittance enhancement effects, with transmittances of 90.7% and 89.3%, respectively. Meanwhile, the PPFC 150 nm/CPI sample exhibited lower transmittance 83.40% than the CPI sample 84.95% in the range of 500 nm and below. However, in the wavelength range beyond 500 nm, the PPFC 150 nm/CPI sample exhibited higher transmittance 91.41% than CPI sample 89.04%, resulting in a higher overall transmittance 89.38% than CPI film 88.00% in the main absorption range of perovskite solar cells, which is 400–800 nm. Figure 4(d) shows the refractive indices of PPFC, CPI, and IGTO, which constitute the bottom part of the solar cell. In the visible region, the refractive indices gradually increased in the order of PPFC, CPI, and IGTO, which are in the order in which light enters, as shown in Figure 4(a). This gradual increase in the refractive index offers the advantage of smooth conversion from the low refractive index of air to higher values, enabling the effective suppression of reflection over a wide range of wavelengths [29,41]. Figure 4(f) shows the transmittance of the substrate on which the PPFC and IGTO were deposited on both sides of the CPI. The enhancement in transmittance owing to the deposition of PPFC was confirmed. For the sample with a 110-nm-thick PPFC layer, which showed the highest effect, the average transmittance in the 400–800 nm range increased by 1.2%, reaching 77.6%, compared to the sample without deposition. In particular, the transmittance at 550 nm increased by 1.6% to 82.5%. And the reflectance data of CPI and CPI/IGTO substrates with and without PPFC deposition are presented in Figure S3, it is confirmed that there is a decrease in reflectance of the substrate with PPFC deposition. Thus, the PPFC thin film was shown to be effective as an ARC layer for FPSCs with CPI and IGTO electrodes.

**Figure 5.**
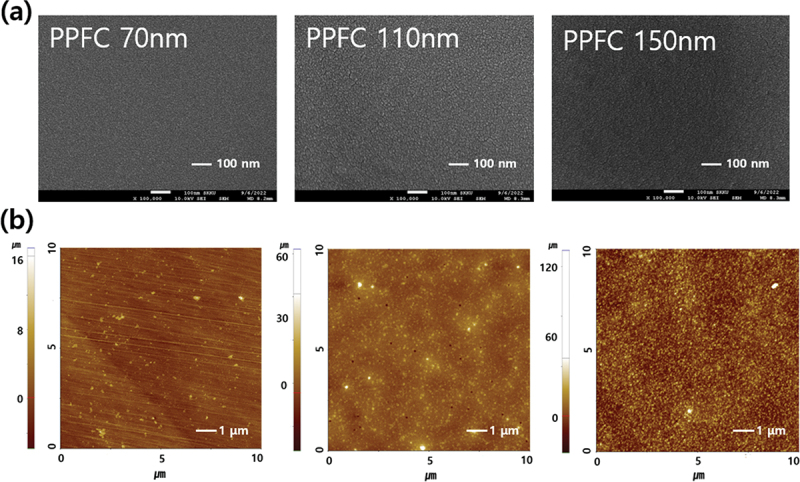
Surface (a) SEM and (b) AFM images of sputtered PPFC sample coated under CPI substrate as a function of thickness.

Figure 5 shows the surface morphology of PPFC thin films as a function of their thickness. SEM analysis Figure 5(a) shows that the surfaces of all the samples with PPFC films are uniform and smooth regardless of film thickness. The PPFC film with a thickness of 70 nm appears to have finer grains, while the ones with thicknesses of 110 nm and 150 nm do not exhibit significant differences. Figure 5(b) shows the results of the AFM analysis. The 3D images in Figure S4 show that as the deposition thickness of PPFC increases from 70 to 150 nm, the height of surface peaks gradually increases. The root mean square (RMS) values for the PPFC films with thicknesses of 70, 110, and 150 nm are 6.8, 31.3, and 29.3 nm, respectively. When the deposition thickness of the PPFC film was 70 nm, the RMS value was small. However, for the 110-nm and 150-nm films, the RMS values were about 5 times higher than the RMS value of the 70-nm film. In the samples with PPFC thicknesses of 110 nm and 150 nm, the difference in roughness was not significant. According to previous research, the transmittance of a sample varies with surface roughness. Therefore, enhancing transmittance by controlling roughness through the formation of nanostructures has been attempted in previous studies [29,41–45]. However, in this study, by observing the difference in transmittance between the PPFC films with thicknesses of 110 nm and 150 nm and similar roughness, the impact of the thickness of the ARC layer on transmittance is speculated to be greater than that of the difference in roughness. However, if the size of the protrusions on the surface or the aspect ratio of their width to height changes, the impact of roughness on transmittance is anticipated to vary [44,45].

**Figure 6.**
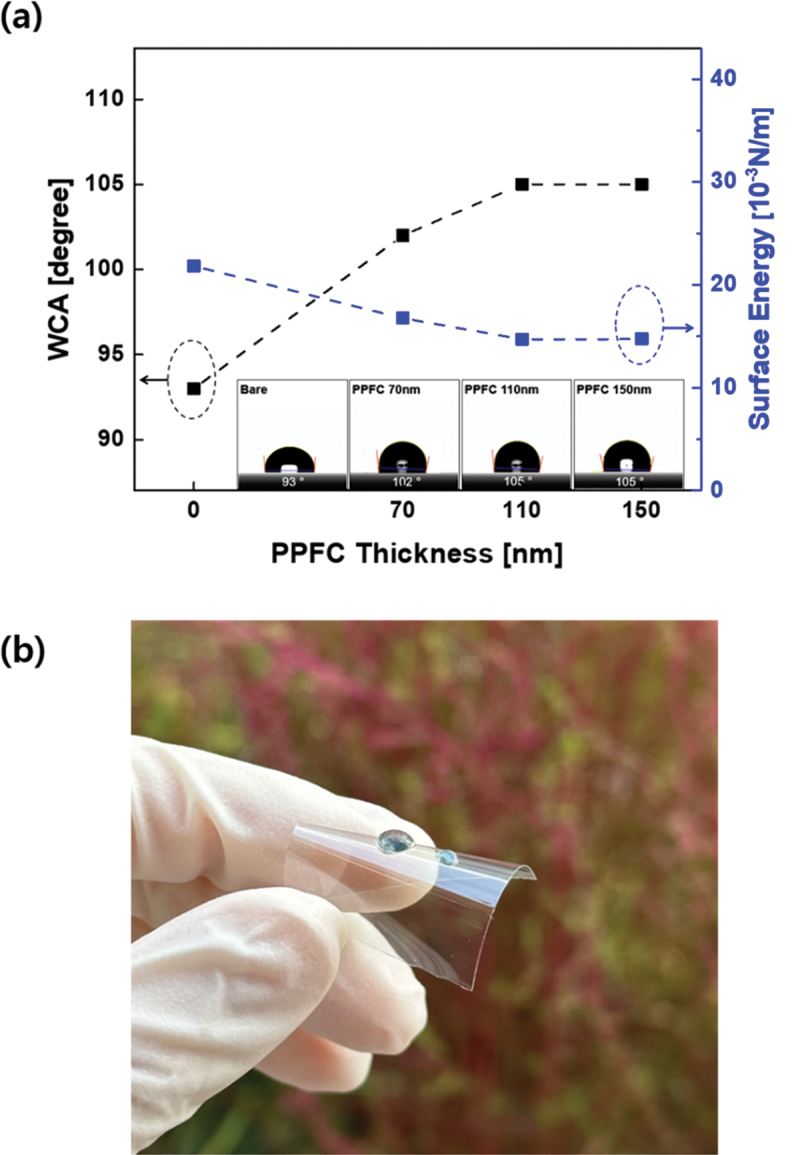
(a) Water contact angle according to PPFC thickness. (b) Photograph of water drop on PPFC thin film.

Surface roughness also influences the wetting characteristics of the surface. Figure 6(a) shows the water contact angle (WCA) values according to the PPFC film thickness. If water droplets are formed on the bottom part of the solar cell substrate owing to rain, they can block or scatter the light entering the solar cell. Therefore, if the bottom side of the substrate possesses hydrophobic characteristics, it can have a positive impact on the solar cell performance. Therefore, the water contact angle (WCA) value of the ARC layer is an important factor. For the CPI substrate without a PPFC layer, the WCA value was measured to be 93°. For the sample with a 70-nm-thick PPFC layer, the WCA value was 102°, whereas for the samples with PPFC thicknesses of 110 and 150 nm, it was 105°.

The WCA value depends on the chemical functional groups and surface roughness. As shown in Figure 4(b), PPFC contains functional groups such as C-F, C-F_2_, C-F_3_, and C-F_n_, which could contribute to reducing the surface energy, thereby imparting hydrophobicity to the PPFC [46]. In addition, the increase in the WCA values as the deposition thickness of PPFC increased from 70 nm to 110 nm and 150 nm is thought to be influenced by surface roughness. When the surface roughness is significant, empty spaces exist between the surface peaks. According to the Cassie-Baxter model, if the empty spaces between the peaks of the hydrophobic surface are filled with air, the water droplets can be lifted upward, resulting in an increase in the contact angle [47–49]. Figure 6(b) shows the hydrophobicity of the PPFC layer. The water droplets adhering to the PPFC layer surface appeared to be nearly spherical in shape. The hydrophobicity of the ARC films induces a self-cleaning effect, aiding in the removal of external contaminants that interfere with light absorption. Blocking moisture from inside a solar cell, which is vulnerable to moisture, can have a positive impact on durability.

**Figure 7.**
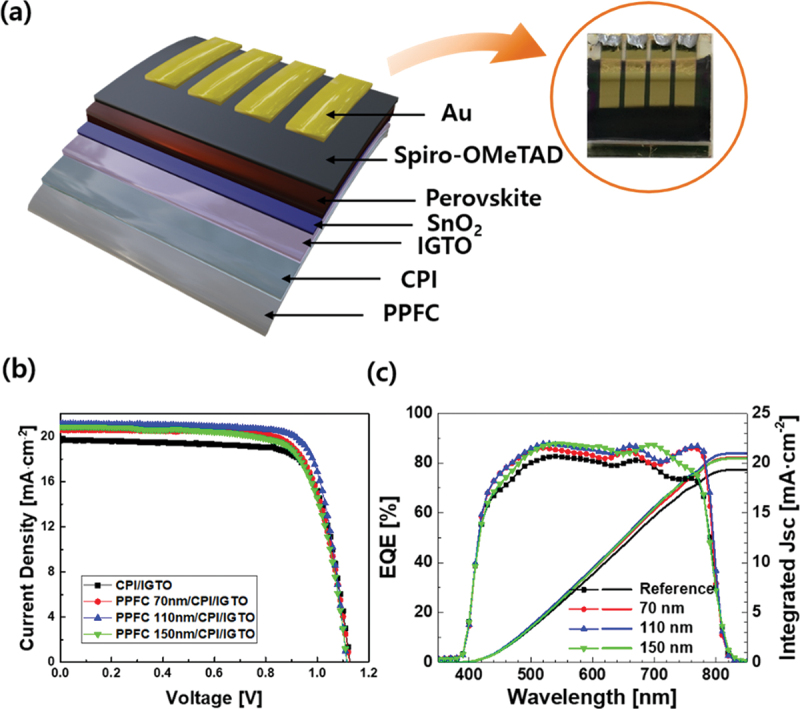
(a) Schematic structure and picture of flexible perovskite solar cell on PPFC/CPI/IGTO substrate. (b) Current density-voltage curves and (c) EQE curves of FPSCs.

To investigate the impact of applying the PPFC/CPI/IGTO structure to solar cells on their properties, we fabricated a solar cell with the structure shown in Figure 7(a): PPFC/CPI/IGTO/SnO2/FA_0.95_MA_0.95_Pb(IBr)_3_/Spiro-OMeTAD/Au. The solar cells were fabricated on a flexible substrate with an area of 2 × 2 cm^2^, and the area of each cell with Au electrodes deposited is 0.096 cm^2^. And the solar cell efficiencies were measured based on the presence or absence of PPFC, and the results are presented in Figure 7(b) and Table 2. The highest efficiency was observed when the PPFC thickness was 110 nm. Particularly noteworthy is the increase in current density to 21.12 mA/cm^2^ compared to that of the solar cell without PPFC, which exhibited a current density of 19.64 mA/cm^2^. Additionally, solar cells with PPFC thicknesses of 70 nm and 150 nm showed current densities of 20.58 mA/cm^2^ and 20.88 mA/cm^2^, respectively, confirming the impact of PPFC deposition on the increase in current density. In addition, the EQE was measured, as shown in Figure 7(c). The results confirmed an increase in EQE due to the deposition of PPFC. The current density values obtained from the EQE results were 19.3 mA/cm^2^ when PPFC was absent and 20.5, 21.0, and 20.5 mA/cm^2^ when the PPFC thickness was 70, 110, and 150 nm, respectively. The current densities measured from an additional 40 fabricated solar cell devices under each condition are shown in Figure S5. It was confirmed that the increased transmittance due to PPFC deposition influenced the increase in current density.

**Table 2.** The highest photovoltaic properties of FPSCs with or without PPFC.

Sample	Voc [V]	Jsc [mA/cm^2^]	FF [%]	PCE [%]
Reference	1.13	19.64	75.4	16.70
PPFC 70 nm	1.13	20.58	74.6	17.27
PPFC 110 nm	1.12	21.12	77.7	18.30
PPFC 150 nm	1.12	20.88	73.1	17.04

**Figure 8.**
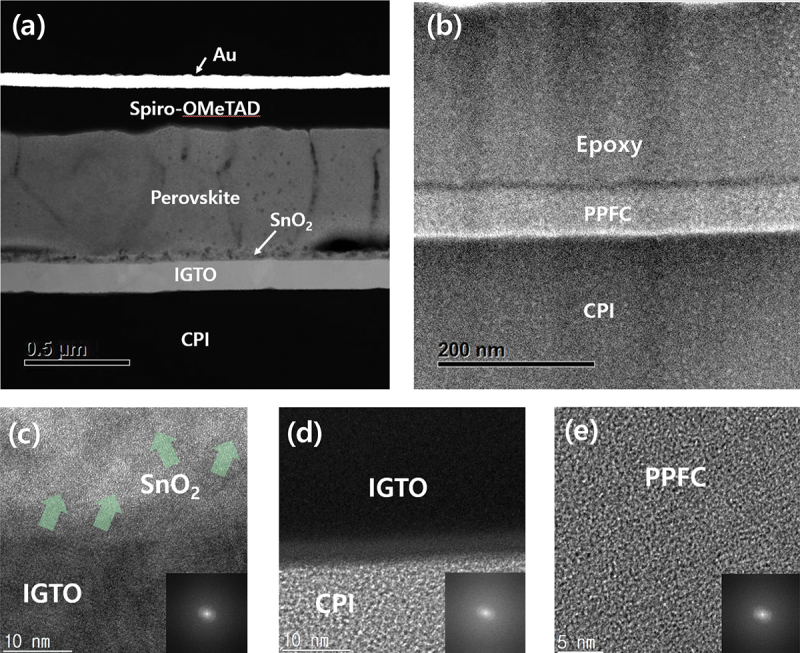
Cross-sectional TEM image of (a) flexible perovskite solar cell and (b) antireflective coating on CPI substrate. Enlarged images and FFT images of (c) IGTO electrode, (d) CPI substrate, and (e) PPFC antireflective layer.

Figures 8(a,b) present the HR-TEM analysis results showing the cross-sectional image of an FPSC. Figure 8(a) shows a cross-sectional image of the upper part of the CPI substrate within an FPSC, and Figure 8(b) shows an image of the ARC layer at the bottom of the CPI substrate. These images confirm that each layer was deposited uniformly. The clear boundaries observed between each layer indicate that no mutual diffusion occurred at the interfaces, and the deposition was well executed without any defects such as voids. Figures 8(c), (d), and (e) show magnified images of each layer, and the fast Fourier transform (FFT) pattern images, displayed in the lower right corner of these images, reveal that the CPI, IGTO, and PPFC layers are all amorphous, unlike SnO_2_, in which the directionality of the crystal is evident. Crystalline thin films scatter the incident light within the crystal lattice, whereas amorphous thin films do not exhibit such scattering, resulting in higher transparency. Therefore, the amorphous property can positively impact the efficiency of solar cells by allowing light to transmit effectively to the photoactive layer. Additionally, the amorphous characteristic offers greater flexibility compared to crystalline films, enhancing the bending durability of FPSCs, making them suitable for constructing the bottom structure of solar cells. These characteristics are thought to be the reason behind the small bending radius and high fatigue durability shown in Figure 3.

## 4. Conclusion

To increase the durability and efficiency of flexible perovskite solar cells, the characteristics of flexible substrates, flexible electrodes, and antireflective coatings were analyzed. For this purpose, a high-temperature-resistant and highly transparent CPI film was used as the flexible substrate. IGTO was deposited on the substrate to serve as a flexible electrode. It has advantageous electrical properties characterized by low resistance, high mobility, flexibility, and high transparency due to its amorphous nature. To enhance the amount of incident light in the photoactive layer, PPFC was applied as an antireflective coating. PPFC showed a low refractive index of 1.38, exhibiting a 3.7% increase in transmittance upon deposition onto CPI compared with its pre-deposition state. Furthermore, the sequential increase in the refractive indices of PPFC, CPI, and IGTO effectively mitigated reflection as light traveled through them in the order of increasing refractive indices. An analysis of the photoelectric conversion efficiency of the device showed that the deposition of PPFC led to an increase in the current density, thus substantiating its effect. The PPFC surface exhibited hydrophobic properties, enabling the self-cleaning of FPSCs, which is expected to have a positive impact on the efficiency of FPSCs. With these characteristics, the PPFC/CPI/IGTO structure was confirmed to be a better option for the bottom structure of flexible solar cells, offering potential for performance enhancement.

## Supplementary Material

Supplemental Material
