# Supplementary material for: Highly flexible and transparent colorless polyimide substrate sandwiched between plasma polymerized fluorocarbon and InGaTiO for high performance flexible perovskite solar cells
Source: Sci Technol Adv Mater. 2024 Aug 6;25(1):2373041. doi: 10.1080/14686996.2024.2373041 (PMC11338216; doi:10.1080/14686996.2024.2373041)
Supplement: Supplemental Material [file TSTA_A_2373041_SM1867.docx]

**Supporting Information**

**Highly flexible and transparent colorless polyimide substrate sandwiched plasma polymerized fluorocarbon and InGaTiO for high performance flexible perovskite solar cells**

Su-Kyung Kim^a^, Eun-Mi Cho^b^, Hae-Jun Seok^a^, Young-Yun Kim^b^, Dong-Hyeok Choi^a,^, Sang-Jin Lee^b^, Nam Joong Jeon^b^, and Han-Ki Kim^a,*^

^a^School of Advanced Materials Science and Engineering, Sungkyunkwan University, Suwon-si, Gyeonggi-do, Republic of Korea

^b^Chemical Materials Solution Center, Korea Research Institute of Chemical Technology, Daejeon, Republic of Korea

Corresponding author: Tel/Fax: +82 31 290 7391/+82 31 290 7410

E-mail address: hankikim@skku.edu (Prof. H.-K. Kim)


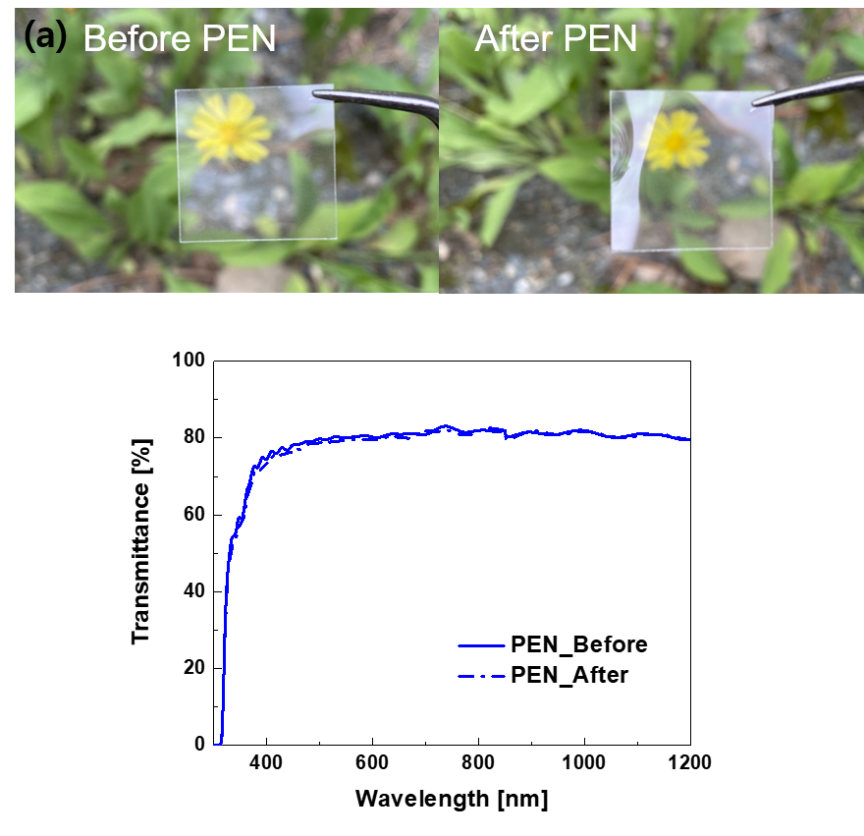


**Figure S1.** (a) Photographs of PEN substrate before and after heat treatment at 180 ℃ for 10 min. (b) Optical transmittance change of PEN substrate before and after heat treatment.


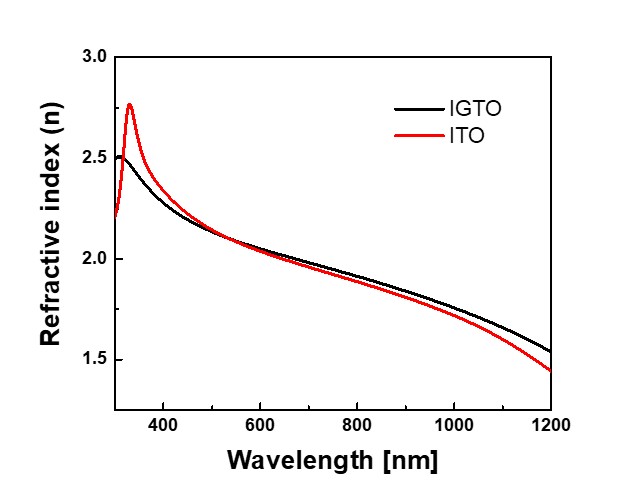


**Figure S2.** Refractive index of IGTO and ITO


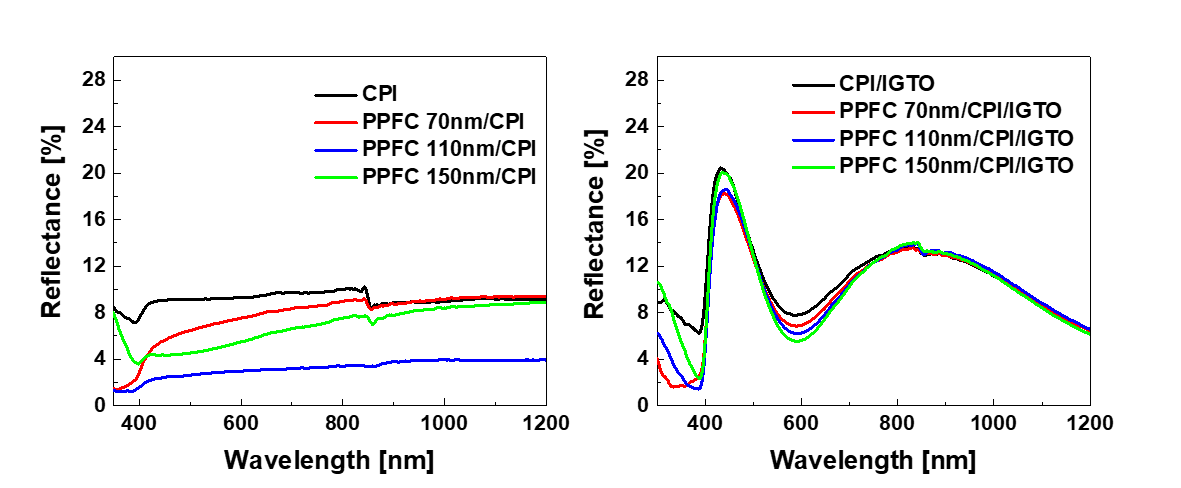


**Figure S3.** Reflectance spectra of (a) PPFC/CPI and (b) PPFC/CPI/IGTO flexible substrate


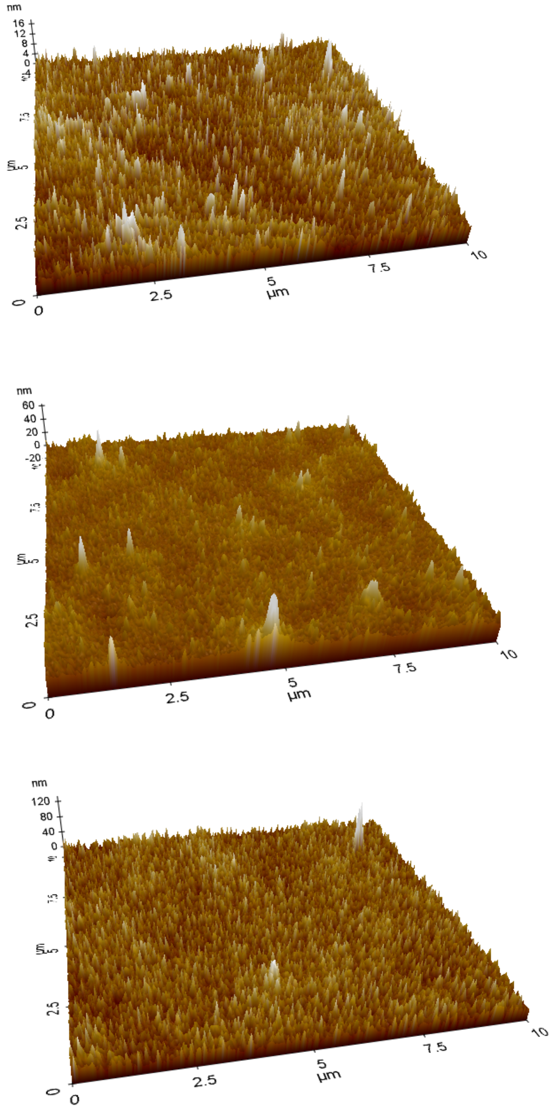


**Figure S4.** AFM 3D images according to PPFC thickness


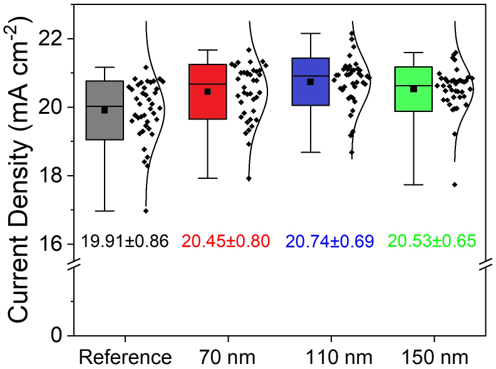


**Figure S5.** Current density distribution of flexible perovskite solar cells with different thickness of the PPFC layer
